# Hyperuricemia has increased the risk of progression of chronic kidney disease: propensity score matching analysis from the KNOW-CKD study

**DOI:** 10.1038/s41598-019-43241-3

**Published:** 2019-04-30

**Authors:** Tae Ryom Oh, Hong Sang Choi, Chang Seong Kim, Eun Hui Bae, Seong Kwon Ma, Su-Ah Sung, Yong-Soo Kim, Kook Hwan Oh, Curie Ahn, Soo Wan Kim

**Affiliations:** 1 Department of Internal Medicine, Chonnam National University Medical School, Gwangju, Korea; 2 Department of Internal Medicine, Eulji University, Seoul, Korea; 3 Department of Internal Medicine, The Catholic University of Korea, Seoul St. Mary’s Hospital, Seoul, Korea; 4 Department of Internal Medicine, Seoul National University, Seoul, Korea

**Keywords:** Predictive markers, Chronic kidney disease

## Abstract

The prevalence of hyperuricemia and chronic kidney disease (CKD) has been steadily increasing. The role of hyperuricemia and efficacy of uric acid-lowering agents against CKD progression remain controversial. This study aimed to evaluate the effect of hyperuricemia and uric acid-lowering agents on the progression of CKD. A total 2042 patients with CKD were analyzed in the KoreaN cohort Study for Outcomes in patients With Chronic Kidney Disease (KNOW-CKD), a prospective cohort study. Patients were classified into quartiles on the basis of their serum uric acid level and the prevalence of advanced CKD was higher in patients with a high uric acid level. A composite renal outcome was defined as one or more of the following: initiation of dialysis or transplantation, a two-fold increase in baseline serum creatinine levels, or a 50% decline in the estimated glomerular filtration rate during the follow-up period. A Cox proportional hazard ratio model was applied to analyze the relationship between composite renal outcome and uric acid levels. The risk of progression to renal failure increased by 28% (hazard ratio [HR], 1.277; 95% confidence interval [CI], 1.212–1.345) for each 1 mg/dl increase in the baseline uric acid level. In multivariate models, an association was found between the highest quartile of uric acid and increased risk of composite renal outcome (HR, 3.590; 95% CI, 2.546–5.063). A propensity score matching analysis was performed to survey the effect of uric acid lowering agent. Both allopurinol and febuxostat did not affect the renal outcome. In conclusion, hyperuricemia appears to be an independent risk factor for composite renal outcome, but allopurinol and febuxostat did not show reno-protective effect.

## Introduction

Uric acid, a final oxidation metabolite of purine in humans, is presumed to have an antioxidant effect and is mainly excreted in urine^1^. Various factors affect the serum uric acid levels, including diuretics (thiazide, furosemide), antihypertensive drugs related to the renin–angiotensin–aldosterone system (RAAS), and daily dietary intake. Studies to clarify the role of uric acid in hypertension, obesity, and insulin resistance, which causes endothelial dysfunction, activation of the RAAS, inflammation, and oxidative stress, have been conducted^2–7^. However, conflicting results on renal outcomes have been reported in humans with and without chronic kidney disease (CKD). Using data from the Chronic Renal insufficiency Cohort clinical trial, Srivastava *et al*.^8^ demonstrated a J-shaped association between hyperuricemia in CKD and mortality as well as higher risk for CKD. Weiner *et al*.^9^ reported that elevated serum uric acid level is a modest, independent risk factor for incident kidney disease in the general population. Krishnan *et al*.^10^ showed that male veterans with gout and serum uric acid levels >7 mg/dl had an increased incidence of kidney disease. In contrast, Kim *et al*.^11^ analyzed the effect of hyperuricemia in patients with end-stage renal disease and found an association between higher uric acid level and lower all-cause mortality and no significant relationship with cardiovascular mortality. Moreover, Chini *et al*.^12^ showed that asymptomatic hyperuricemia was not an independent risk factor for CKD progression. Chonchol *et al*.^13^ reported that no significant association was found between uric acid level and incident CKD. Madero *et al*.^14^, in a study of patients with stages 3 to 4 CKD, demonstrated that hyperuricemia appears to be an independent risk factor for all-cause and cardiovascular mortality, but not kidney failure. Distinguishing the exact effect of serum uric acid levels on CKD progression is of great importance. If uric acid is an independent risk factor associated with CKD, it will be a modifiable risk factor that can be relatively easily corrected. Therefore, this study aimed to determine the correlation between serum uric acid levels and CKD progression and to identify the role of uric acid-lowering agents through analysis of the data of the KNOW-CKD study.

## Results

### Clinical characteristics of the study population

**Figure 1:**
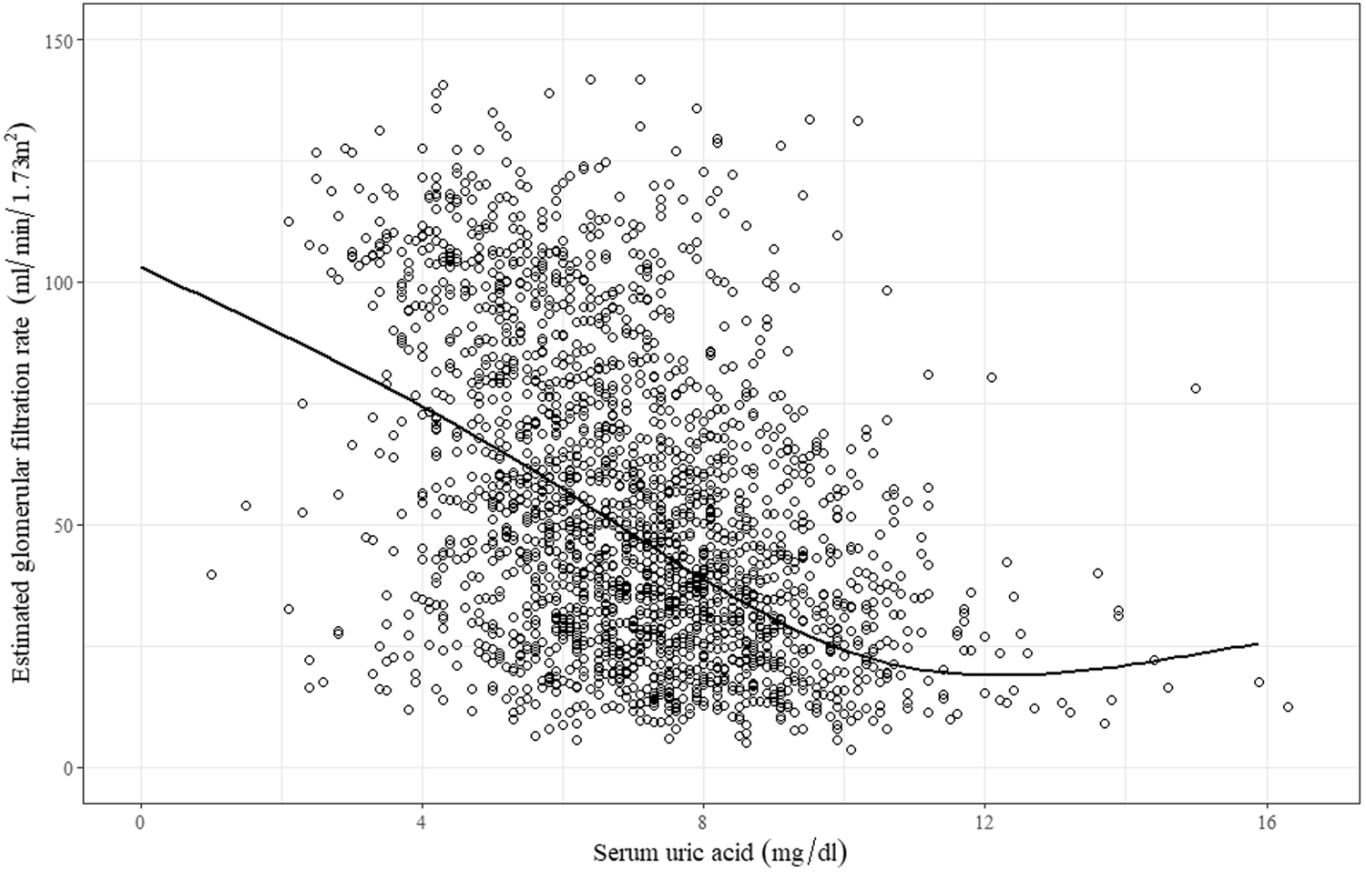
Correlation between estimated glomerular filtration rate and serum uric acid. As the CKD progressed, the uric acid level tended to increase.

Table 1 shows a summary of the clinical characteristics of the enrolled patients, for all subjects and the quartile groups. The median duration of follow-up was 2.12 [interquartile range, 1.02:3.81] years. The mean ages at the time of enrollment were 56 years and 53 years for male and female patients, respectively, and 38.5% of the patients were female. The mean serum uric acid level was 7.01 ± 1.91 mg/dl, and the mean estimated glomerular filtration rate (eGFR) was 52.8 ml/min per 1.73 m^2^. Participants with higher uric acid levels were more likely to be male, had a higher prevalence of diabetes (DM) (p = 0.002), and tended to take more uric acid-altering medications, including thiazide or loop diuretics, angiotensin-converting enzyme (ACE) inhibitors, angiotensin receptor blockers (ARBs), allopurinol, and febuxostat medications (Table 1). The patients with higher uric acid levels had lower eGFR (p < 0.001). Figure 1 presents their correlation.

**Table 1:** Clinical characteristics of the subjects stratified by baseline serum uric acid categories.

	All subjects	Hyperuricemia groups				
		Quartile 1	Quartile 2	Quartile 3	Quartile 4	p-trend
Age (years)	53.8 ± 12.1	52.2 ± 11.8	54.6 ± 11.9	54.7 ± 11.9	54.0 ± 12.8	0.002
Female (n(%))	787 (38.5)	291 (54)	200 (39.2)	163 (33.2)	133 (26.5)	0.000
SBP (mmHg)	128	126	128	127	128	0.044
DBP (mmHg)	77	77	78	77	76	0.044
MBP (mmHg)	94	93	95	94	93	0.206
DM (n(%))	683 (33.5)	151 (28.1)	161 (31.6)	178 (36.3)	193 (38.5)	0.002
BMI	24.51 ± 3.36	24.07 ± 3.44	24.57 ± 3.42	24.72 ± 3.26	24.71 ± 3.36	0.004
CHF (n(%))	28 (1.4)	7 (1.3)	5 (1.0)	6 (1.2)	10 (2.0)	0.550
CVD (n(%))	126 (6.2)	24 (4.5)	30 (5.9)	36 (7.3)	36 (7.2)	0.184
PVD (n(%))	73 (3.6)	17 (3.2)	15 (2.9)	22 (4.5)	19 (3.8)	0.551
**Laboratory**						
Uric acid (mg/dL)	7.01 ± 1.91	4.73 ± 0.82	6.40 ± 0.37	7.63 ± 0.35	9.51 ± 1.15	0.000
Hemoglobin (g/dL)	12.8 [11.3; 14.3]	13.1 [11.9; 14.3]	13.0 [11.4; 14.6]	12.7 [11.1; 14.3]	12.2 [10.9; 14.1]	0.000
Creatinine (mg/dL)	1.3 [1.0; 1.8]	1.0 [0.8; 1.4]	1.3 [1.0; 1.6]	1.4 [1.1; 1.9]	1.5 [1.2; 2.2]	0.000
CKD-EPI creatinine equation (ml/min/1.73 m^2^)	52.80 ± 30.46	71.39 ± 32.41	55.28 ± 28.67	45.26 ± 25.66	37.72 ± 22.60	0.000
HbA1c (n(%))	6.5 [5.8; 7.5]	6.4 [5.7; 7.4]	6.4 [5.7; 7.8]	6.6 [5.9; 7.3]	6.6 [5.8; 7.5]	0.705
Albumin (g/dL)	4.18 ± 0.42	4.23 ± 0.43	4.17 ± 0.42	4.15 ± 0.40	4.16 ± 0.42	0.006
Total cholesterol (mg/dL)	171.0 [146.0; 197.0]	176.0 [154.0; 198.0]	172.5 [146.0; 198.0]	167.0 [145.0; 199.0]	164.0 [138.0; 191.0]	0.000
Low-density lipid (mg/dL)	93.0 [73.0; 115.0]	95.0 [78.0; 116.0]	94.0 [73.0; 117.0]	92.0 [74.0; 114.5]	88.0 [69.0; 112.0]	0.004
High-density lipid (mg/dL)	46.8 [38.0; 57.0]	51.0 [41.0; 63.2]	47.0 [39.0; 57.0]	45.0 [37.0; 55.0]	42.0 [36.0; 52.0]	0.000
Triglyceride (mg/dL)	133.0 [92.0; 194.0]	119.0 [82.0; 172.0]	131.0 [93.0; 194.0]	140.0 [98.0; 198.5]	143.0 [96.0; 207.0]	0.000
hsCRP (mg/dL)	0.6 [0.2;1.7]	0.5 [0.2; 1.3]	0.6 [0.3; 1.7]	0.7 [0.3; 1.8]	0.8 [0.3; 2.1]	0.000
Calcium (mg/dL)	9.12 ± 0.53	9.17 ± 0.49	9.15 ± 0.50	9.09 ± 0.56	9.06 ± 0.57	0.001
Phosphate (mg/dL)	3.69 ± 0.67	3.57 ± 0.54	3.58 ± 0.63	3.73 ± 0.70	3.87 ± 0.76	0.000
tCO_2_ (mmol/L)	26.0 [23.0; 28.0]	27.0 [24.0; 29.0]	26.0 [24.0; 28.0]	25.0 [23.0; 28.0]	25.0 [22.1; 27.0]	0.000
UPCR (mg/mgCr)	0.5 [0.2; 1.5]	0.3 [0.1; 0.8]	0.5 [0.1; 1.5]	0.7 [0.3; 1.8]	0.6 [0.2; 2.0]	0.000
**Medication**						
ACEi (n(%))	227 (11.1)	60 (11.1)	46 (9)	53 (10.8)	68 (13.6)	0.146
ARB (n(%))	1653 (81)	406 (75.3)	414 (81.2)	413 (84.1)	420 (83.8)	0.001
Diuretics (n(%))	657 (32.2)	99 (18.4)	128 (25.1)	183 (37.3)	247 (49.3)	0.000
Allopurinol (n(%))	295 (14.5)	37 (6.9)	80 (15.7)	100 (20.4)	78 (15.6)	0.000
Febuxostat (n(%))	108 (5.3) c	68 (12.6)	17 (3.3)	6 (1.2)	17 (3.4)	0.000

Abbreviations: SBP, systolic blood pressure; DBP, diastolic blood pressure; MBP, mean arterial blood pressure; DM, diabetes mellitus; BMI, body mass index; CHF, congestive heart failure; CVD, cerebrovascular disease; PVD, peripheral vascular disease; HbA1c, hemoglobin A1c; hsCRP, high sensitivity C-reactive protein; tCO_2_, total CO_2_; UPCR, urine protein–creatinine ratio; ACEi, angiotensin-converting enzyme inhibitor; ARB, angiotensin receptor blocker.

### Uric acid quartiles and composite renal outcome

**Figure 2:**
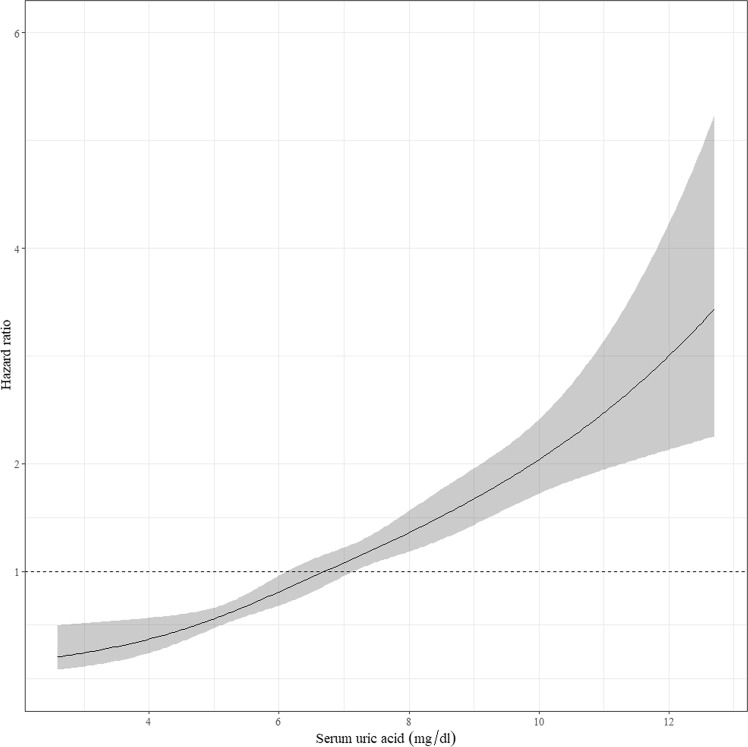
Cubic spline curve for Cox proportional hazard model of serum uric acid. A linear relationship between renal outcome and uric acid levels was observed.

A total of 340 (16.7%) patients experienced composite renal outcome during the follow-up period. The composite renal outcome occurred in 43 in quartile 1, 67 in quartile 2, 95 in quartile 3, and 135 in quartile 4 (p for trend <0.001). In multivariable adjusted model, the risk of composite renal outcome increased by 35% (hazard ratio [HR], 1.347; 95% confidence interval [CI], 1.171–1.549; p < 0.001) for every 1 mg/dL increase in baseline uric acid level. Patients in quartile 4 had significantly higher HR of composite renal outcome than those in quartile 1 (HR, 2.464; 95% CI, 1.119–5.426; p = 0.003; Table 2). Figure 2 shows a linear relationship between renal outcome and uric acid levels.

**Table 2:** Cox proportional hazard ratio model for hyperuricemia and composite renal outcome in all subjects by uric acid level and quartiles of uric acid.

	Composite renal outcome			Doubling of creatinine or 50% decline of eGFR			Initiation of dialysis or transplantation		
	Unadjusted HR (95% CI)	Adjusted HR (95% CI)	Incidence	Unadjusted HR (95% CI)	Adjusted HR (95% CI)	Incidence	Unadjusted HR (95% CI)	Adjusted HR (95% CI)	Incidence
Uric acid	1.277 (1.212–1.345)	1.347 (1.171–1.549)	—	1.196 (1.109–1.290)	0.990 (0.895–1.096)	—	1.311 (1.238–1.388)	1.535 (1.314–1.793)	—
Male	1.321 (1.224–1.425)	1.164 (1.072–1.265)	—	1.243 (1.107–1.396)	1.134 (1.001–1.286)	—	1.323 (1.217–1.438)	1.170 (1.070–1.278)	—
Female	1.312 (1.200–1.434)	1.175 (1.050–1.315)	—	1.238 (1.095–1.399)	1.191 (1.021–1.390)	—	1.389 (1.259–1.533)	1.256 (1.103–1.430)	—
Quartile 1	1 (reference)	1 (reference)	43 (12.6%)	1 (reference)	1 (reference)	30 (17.5%)	1 (reference)	1 (reference)	30 (10.7%)
Quartile 2	1.708 (1.165–2.506)	1.002 (0.434–2.316)	67 (19.7%)	1.255 (0.768–2.051)	0.653 (0.360–1.187)	34 (19.9%)	1.892 (1.207–2.965)	1.544 (0.499–4.774)	52 (18.6%)
Quartile 3	2.459 (1.715–3.525)	1.206 (0.479–3.041)	95 (27.9%)	2.021 (1.293–3.159)	0.941 (0.541–1.639)-	54 (31.6%)	2.957 (1.945–4.495)	3.102 (0.973–9,885)	81 (28.9%)
Quartile 4	3.590 (2.546–5.063)	2.464 (1.119–5.426)	135 (39.7%)	2.073 (1.324–3.248)	0.795 (0.441–1.434)	53 (31.0%)	4.469 (2.990–6.678)	6.015 (2.020–17.913)	117 (41.8%)

Note: CKD progression is adjusted for calcium, creatinine, total CO_2_, hemoglobin, uric acid, phosphate, and urine protein–creatinine ratio.

Initiation of dialysis or transplantation is adjusted for age, sex, calcium, creatinine, total CO_2_, total cholesterol, low-density lipid, high-density lipid, triglyceride, hemoglobin, uric acid, phosphate, and urine protein–creatinine ratio.

Abbreviations: HR, hazard ratio; CI, confidence interval; eGFR, estimated glomerular filtration rate.

### Subgroup analysis

The HRs (95% CIs) of sex-specific Cox proportional hazard model were 1.164 (1.072–1.265) in men and 1.175 (1.050–1.315) in women. Regardless of sex, serum uric acid levels had a major influence on the composite renal outcome (Table 2). The HRs for the subcategories of composite renal outcome, initiation of dialysis or kidney transplantation or a twofold increase in baseline serum creatinine or 50% decline of estimated glomerular filtration rate during the follow-up period were analyzed. Table 2 shows the results of multivariable adjusted Cox proportional hazard model. Serum uric acid level was not an independent risk factor for the doubling of creatinine or a half decline of eGFR. However, a strong association was found between serum uric acid level and the initiation of dialysis or kidney transplantation (HR, 1.535; 95% CI, 1.314–1.793; p < 0.001).

**Figure 3:**
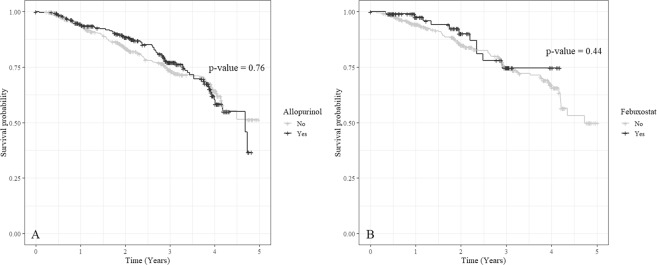
Kaplan-Meier curve with log-rank test for renal outcome by uric acid lowering agents. (**A**) Allopurinol, (**B**) Febuxostat.

The effects of uric acid-lowering agents such as febuxostat and allopurinol on composite renal outcome were analyzed. Propensity score matching analysis was performed because significant differences were observed in the number of patients and clinical characteristics in the medication- and non-treated groups. The variables used as covariates were as follows: age, sex, eGFR, presence of diabetes mellitus, mean arterial blood pressure for hypertension, serum albumin and proteinuria. Matching using optimal matching method was conducted, and the standardized difference of covariates was reviewed. The standardized difference of all covariates was below 0.1. In the matching process, the missing values were excluded. Before matching, 295 patients were treated with allopurinol and 108 were treated with febuxostat. After matching, the numbers of allopurinol- and febuxostat-treated patients were 281 and 99, respectively. The characteristics of allopurinol and febuxostat groups were uniformly controlled, but the Kaplan-Meier curve with log rank test did not show any statistical significance in both groups (Fig. 3). Tables 3 and 4 summarizes the before and after result of propensity score matching analysis.

**Table 3:** Propensity score matching analysis for the allopurinol-treated group.

Variables		Before matching				After matching			
		(−) allopurinol	(+) allopurinol	P-value	Standardized difference	(−) allopurinol	(+) allopurinol	P-value	Standardized difference
Uric acid (mg/dL)		6.95 ± 1.95	7.39 ± 1.59			7.46 ± 1.92	7.42 ± 1.55		
**Covariates**									
Age (years)		53.3	56.7	<0.001	0.286	57.1	56.7	0.636	0.034
Sex (n)	Male	1025 (58.7%)	229 (77.6%)	<0.001	0.415	438 (77.94%)	219 (77.94%)	1.000	0.000
	Female	721 (41.3%)	66 (22.4%)			124 (22.06%)	62 (22.06%)		
Diabetes (n.)	Yes	611 (35.1%)	72 (24.4%)	<0.001	0.235	414 (73.67%)	214 (76.16%)	0.485	0.057
	No	1132 (64.9%)	223 (75.6%)	<0.001		148 (26.33%)	67 (23.84%)		
eGFR (ml/min/1.73 m^2^)		55.2 ± 31.3	38.8 ± 20.3	<0.001	0.620	39.20 ± 21.11	38.76 ± 20.47	0.772	0.021
MBP (mmHg)		93.7	93.8	0.953	0.004	94.6	94.4	0.773	0.021
Albumin (mg/dL)		4.18 ± 0.43	4.19 ± 0.38	0.670	0.026	4.19 ± 0.38	4.19 ± 0.38	0.849	0.014
Proteinuria (mg/gCr)		118.5 ± 182.8	89.6 ± 117.3	0.030	0.188	90.51 ± 127.4	89.87 ± 117.4	0.944	0.005

Abbreviations: MBP, mean arterial blood pressure, eGFR, estimated glomerular filtration rat.

**Table 4:** Propensity score matching analysis for the febuxostat-treated group.

Variables		Before matching				After matching			
		(−) febuxostat	(+) febuxostat	P-value	Standardized difference	(−) febuxostat	(+) febuxostat	P-value	Standardized difference
Uric acid (mg/dL)	7.09 ± 1.85;	5.72 ± 2.36			7.45 ± 1.7	5.82 ± 2.37			
**Covariates**									
Age (years)		53.8 ± 12.1	55.1 ± 12.2	0.262	0.111	56.14 ± 11.58	55.07 ± 12.00	0.416	0.091
Sex (n)	Male	1159 (60.0%)	95 (88.0%)	<0.001	0.673	351 (88.64%)	86 (86.87%)	0.753	0.054
	Female	773 (40.0%)	13 (12.0%)			45 (11.36%)	13 (13.13%)		
Diabetes (n)	Yes	649 (33.6%)	34 (31.5%)	0.720	0.046	136 (34.34%)	32 (32.32%)	0.794	0.043
	No	1280 (66.4%)	74 (68.5%)			260 (65.66%)	67 (67.68%)		
eGFR (ml/min/1.73 m^2^)		53.4 ± 30.9	41.5 ± 19.4	<0.001	0.465	41.66 ± 22.83	41.33 ± 19.93	0.893	0.016
MBP (mmHg)		93.7	93.4	0.742	0.032	93.8	93.5	0.786	0.030
Albumin (mg/dL)		4.17 ± 0.42	4.25 ± 0.37	0.033	0.200	4.23 ± 0.39	4.25 ± 0.32	0.573	0.060
Proteinuria (mg/gCr)		114.9 ± 176.9	102.0 ± 139.2	0.515	0.081	99.27 ± 140.9	102.03 ± 139.2	0.862	0.020

Abbreviations: MBP, mean arterial blood pressure, eGFR, estimated glomerular filtration rat.

## Discussion

Uric acid has been suggested to correlate with various diseases since the 19th century^15^. However, unlike other known risk factors, it is not recognized as an independent risk factor in previous studies. In the case of CKD, renal excretion of uric acid is decreased, resulting in hyperuricemia. It is thought that the interstitial accumulation of sodium urate induces the deterioration of the disease. However, contrary evidence has been suggested, and the causal relationship between uric acid and renal disease has been controversial^16^. Recently, uric acid has been recognized as an independent risk factor and the role of various uric acids has been reconsidered.

Uric acid has varying biological actions^17^. It has antioxidant effects in the extracellular environment^18^, which play an important role in neurologic diseases^19,20^. In addition, uric acid has been identified to have effects on the immune system and proinflammatory pathways. In immune response, uric acid may aid in the recognition of apoptotic cells by dendritic cells and in the activation of CD8 cells^21^. A high intracellular uric acid concentration facilitates protein kinase and transcription of proinflammatory cytokines and chemokines and provokes proximal tubular dysfunction with the release of inflammatory chemokines^22–24^. Likewise, its function is linked to various systems related to the kidneys.

In a recent study, the density and fractal dimension of the tubule through fractal analysis showed a strong positive correlation with eGFR^25^. Uric acid showed a negative correlation, and the authors suggested that it may be a predictive factor for assessing nephron integrity. Including this study, several studies have been conducted to determine whether uric acid is a risk factor of CKD. However, as mentioned above, the role of hyperuricemia in CKD progression still remains controversial^8–10,12–14^.

**Figure 4:**
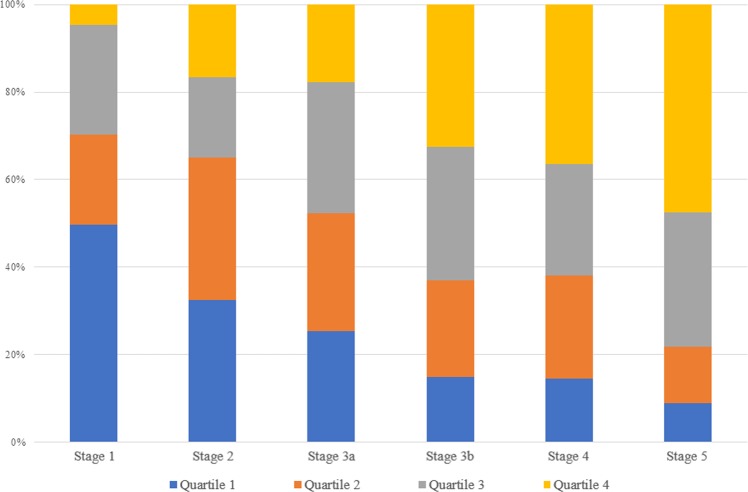
Distribution of CKD stage according to the extent of hyperuricemia.

In the current study, serum uric acid concentration tended to be higher when baseline eGFR was lower (Fig. 1). As expected, this correlation was caused by impaired renal excretion of serum uric acid by a decrease in eGFR. This suggests that patients with renal impairment are more likely to be exposed to higher uric acid concentrations, which means that the multisystem effect of uric acid can be further strengthened. Our study demonstrated that high uric acid was associated with poor renal outcome, and a strong correlation was found between higher uric acid concentrations and the risk associated with initiation of dialysis or kidney transplantation. However, it did not show sufficient statistical significance for 50% decline of eGFR or doubling of baseline creatinine. This result may be caused by an increase in the proportion of patients with higher serum uric acid levels due to progression of CKD (Fig. 4).

There is still debate on the use of uric acid-lowering agents in hyperuricemia. The uric acid-lowering agents can be classified as direct-acting and indirect-acting agents. Direct-acing agents include allopurinol, febuxostat, and rasburicase, and indirect-acting agents include canagliflozain, losartan, fenofibrate, and sevelamer. In general, allopurinol and febuxostat are the most commonly used drugs in hyperuricemia. Febuxostat is a selective inhibitor of non-purine xanthine oxidase. Allopurinol has lower affinity for xanthine oxidase and xanthine dehydrogenase than febuxostat^26^. Therefore, higher doses of allopurinol are necessary to maintain the efficacy of the medication than those of febuxostat. In addition, febuxostat is structurally different from allopurinol. Since febuxostat is structurally different from the purine or pyridine series, it does not have a major effect on purine and pyrimidine metabolism. Additionally, severe adverse effects, such as idiosyncratic allergic reaction, are less likely to occur with febuxostat than with allopurinol. However, febuxostat is more expensive, and the clinician chooses a drug according to the patient’s economic situation. Also, the effect of uric acid-lowering agents on renal outcome has been the topic of several debates. Animal studies showed renoprotective effects of uric acid-lowering agents^27^. However, to date, the effects of uric acid-lowering agents in primary or secondary prevention of renal disease in humans are not fully elucidated^28,29^. In addition, studies examining the differences between uric acid-lowering agents such as allopurinol and febuxostat are very limited. Febuxostat blocks urate production and reduces both intra- and extracellular uric acid concentrations. It may slow the progression of CKD by lowering the serum uric acid concentrations, which reduces oxidative stress and suppression of the endothelial dysfunction and tubular injury through xanthine oxidase inhibition^30,31^. Treatment with febuxostat may prevent glomerular hypertension, afferent arteriolar thickening, and ischemic renal histologic changes and ameliorate renal damage^32^. The renoprotective effect of febuxostat might be expressed by these mechanisms which are modifiable factors for the CKD progression.

We analyzed the effect of uric acid-lowering agent through propensity score matching analysis to minimize the influence of confound variables as much as possible, because this study was not a prospective study. The higher uric acid level of the allopurinol-treated group than the target goal of uric acid level for CKD in both before and after matching was observed. A rare but serious hypersensitivity reaction has been associated with allopurinol, and this reaction is idiosyncratic and unpredictable. For this reason, patients receiving allopurinol tend to be relatively underdosed compared with patients receiving other uric acid-lowering agents, and insufficient titration of allopurinol is often observed^31^. Febuxostat tends to cause fewer side effects and may not require drug dose adjustment according to renal function. In addition, it is more potent than allopurinol and could easily reach the target level of serum uric acid. Although only a few large randomized controlled trials have been conducted, other studies have reported that febuxostat efficacy is better than allopurinol. Chou *et al*.^33^ demonstrated that febuxostat and benzbromarone may be more effective than allopurinol in reducing CKD progression and in lowering serum uric acid levels in patients with CKD. Sezai *et al*.^34,35^, in a study of hyperuricemic patients with cardiac surgery with or without chronic kidney disease, reported that febuxostat is superior to allopurinol in suppressing serum uric acid levels and protecting renal function. Apart from drug efficacy, the use of uric acid-lowering agents did not have any beneficial effects against progression of CKD in the current study (Fig. 3). Propensity score matching was performed appropriately, but allopurinol- and febuxostat-treated groups did not yield a statistically significant outcome. This result is similar to that of the recent FEATHER study, a randomized controlled trial in which febuxostat failed to mitigate CKD progression^36^. However, since the FEATHER study was performed only on patients with stage 3 CKD, this result should be interpreted with caution. In this study, baseline characteristics showed a large difference, and some variables were suggested to act as confounders. For example, sex, age, and baseline uric acid levels were significantly different. In addition, several factors were related to hyperuricemia such as food and medicines. However, due to the limitation of data, these aspects are not fully reflected in this analysis. Additionally, the number of drug-taking patients is smaller than the total number of hyperuricemia patients. Therefore, we could not establish the statistical significance in this study. Further studies are needed to demonstrate the effects of uric acid-lowering agents on CKD progression in patients with all stages of CKD.

This survey has some limitations worth noting. First, although the KNOW-CKD study is planned as a prospective observational study, these results are from an initial cross-sectional study. As the KNOW-CKD study is still in progress, if follow-up data of uric acid levels were sufficient, we could have conducted better analysis of the correlation between renal outcome and uric acid. Second, the previous history of medication for only 1 month before the study was investigated. Therefore, the exact period of taking uric acid-lowering agents was not known. The influence of uric acid-lowering agents on the kidneys, not through the serum uric acid levels, is also a topic of great interest. Moreover, information on the duration of drug use was very limited, and there was a limit to the analysis of the direct or indirect effects of uric acid-lowering agents on the kidneys. Third, information on foods that can affect serum levels of uric acid was not available. Serum uric acid levels are known to be influenced by food. If information on dietary habits is analyzed together, we can deduce better conclusions. Despite these limitations, our study demonstrated the effect of hyperuricemia on renal outcome in pre-dialysis CKD patients. Well-designed randomized controlled trials on the exact impact of uric acid-lowering agents on CKD progression are necessary in the future.

## Methods

### Study population and ethic statement

We reviewed baseline data from the KoreaN Cohort Study for Outcome in Patients With Chronic Kidney Disease (KNOW-CKD), a nationwide multicenter prospective cohort study that included non-dialysis patients with stage 1–5 CKD, aged 20–75 years. The detailed design and methods of the KNOW-CKD study have been previously published (NCT01630486 at http://www.clinicaltrials.gov)^37^. All procedures performed in the participants were in accordance with the ethical standards of the institutional and national research committee at which the studies were conducted (IRB approval number CNUH-2011-092) and with the 1964 Helsinki declaration and its later amendments or comparable ethical standards. The study protocol was approved by the institutional review board at each participating clinical center, as follows: Seoul National University Hospital (1104-089-359), Seoul National University Bundang Hospital (B-1106/129-008), Yonsei University Severance Hospital (4-2011-0163), Kangbuk Samsung Medical Center (2011-01-076), Seoul St. Mary’s Hospital (KC11OIMI0441), Gil Hospital (GIRBA2553), Eulji General Hospital (201105-01), Chonnam National University Hospital (CNUH-2011-092), and Pusan Paik Hospital (11-091) in 2011. Written informed consent was obtained from all study participants. The KNOW-CKD study included 2238 patients. For our analysis, we obtained information on serum uric acid levels and renal outcome from 2042 non-dialysis patients with CKD.

### Data collection and survey instruments

Demographics and laboratory data of baseline were searched by electronic data management system (PhactaX, Seoul, Republic of Korea) with the help of data management department of Seoul National University Medical Research Collaborative Center. Demographic data, including age, sex, comorbid diseases, blood pressure, previous history of medication within 1 month, height, and weight, were collected. Initial laboratory measurements included the following items: hemoglobin, albumin, creatinine, uric acid, total cholesterol, triglyceride, high-density lipoprotein cholesterol (HDL-C), low-density lipoprotein cholesterol (LDL-C), high sensitivity C-reactive protein (hs-CRP), calcium, inorganic phosphate, total CO2, hemoglobin A1c (HbA1c), and urinary protein-to-creatinine ratio. All these venous blood samples were collected after an overnight fast. First-voided urine was used for measurement of spot urinary metrics, such as protein and creatinine. All samples prior to May 1, 2013 were retrospectively analyzed using the samples stored in the Biobank. From May 1, 2013, samples were collected from each institution and measured in real time for samples transferred to the central laboratory. A medication history of one month before enrollment in this study was recorded. The history of the drug included diuretics such as furosemide, thiazide, and spironolactone, allopurinol, febuxostat, ACE inhibitor, and ARB.

### Definition

Hyperuricemia was defined as a plasma uric acid level >7.0 mg/dL by Japanese guideline^38^. Serum creatinine was measured using traceable isotope-dilution mass spectrometry method. The estimated glomerular filtration rate was calculated using the Chronic Kidney Disease Epidemiology Collaboration (CKD-EPI) equation^39^. Composite renal outcome, so-called CKD progression, was defined as one or more of the following: initiation of dialysis or kidney transplantation, a twofold increase in baseline serum creatinine, or 50% decline of estimated glomerular filtration rate (eGFR) during the follow-up period.

### Statistical analysis

The enrolled patients were classified as quartiles based on serum uric acid levels. Among the continuous variables, normal distribution data were expressed as mean with standard deviation, and skewed data were described as median with interquartile range. The Shapiro–Wilk normality test was used for normality test. Description of categorical variables was presented in the number of participants (percentage). One-way analysis of variance was used for normal distributed data, and the Kruskal–Wallis test was used for skewed data to identify the differences and compare clinical characteristics between the groups. The Cochran–Armitage trend test was conducted for categorical variables. Cox proportional hazard regression model was applied to survey the independent risk factors associated with composite renal outcome. Statistically significant variables were identified through backward elimination; then, the multivariable Cox proportional hazard regression model was adjusted for these items by the enter method. HR and 95% CI were calculated to compare the risk of composite renal outcome. To access the mutual influence between variables, collinearity diagnosis was conducted. A propensity score matching analysis was used to evaluate the effect of uric acid-lowering agents on composite renal outcome between the uric acid-lowering agent-treated and the non-treated groups. For propensity score matching analysis, ‘MatchIt’ packages in R language were used^40^, and the standardized difference was reviewed before and after matching. Whether the covariance was corrected properly was confirmed^41^.

Data were analyzed using SPSS version 20 for Windows (IBM Corp., Armonk, NY, USA) and R language (version 3.4.4; R Foundation for Statistical Computing)^42^. *P*-values < 0.05 were considered statistically significant.

## Data Availability

Available as supplementary material when accepted.
